# PReS-FINAL-2196: The clinical significance of the Q703K mutation of NLRP3 gene. A multicentric national study

**DOI:** 10.1186/1546-0096-11-S2-P186

**Published:** 2013-12-05

**Authors:** A Naselli, L Cantarini, A Insalaco, M Alessio, A Tommasini, R Gallizzi, S Signa, OM Lucherini, F Caroli, I Ceccherini, A Martini, M Gattorno

**Affiliations:** 1 Pediatria II, IRCCS, G. Gaslini, Genoa, Italy; 2 Policlinico Le Scotte, University of Siena, Siena, Italy; 3 UO Reumatologia, Ospedale Bambino Gesù, Rome, Italy; 4 Dipartimento di Pediatria, Ospedale Federico II, Naples, Italy; 5 Dipartimento di Pediatria, IRCCS, B. Garofalo, Trieste, Italy; 6 A.O.U. Policlinico G. Martino, Messina, Italy; 7 Laboratorio Genetica Molecolare e Citogenetica, IRCCS G. Gaslini, Genoa, Italy

## Introduction

The Q703K is a variant of NLRP3 gene has an unknown pathogenetic significance. It, has been considered to be a clinically unremarkable polymorphism, due to its presence in 12-20% of general population. However, a recent study has shown that carriers of the Q703K display an higher secretion of IL-1b, thus suggesting a possible pathogenic role of this variant.

## Objectives

to analyse the prevalence of Q703K mutation in patients screened for suspected CAPS and to describe the clinical and biomarkers findings of patients carrying of this mutation.

## Methods

from 2002 the molecular analysis of the NLRP3 gene was performed in 615 patients with a clinical history suggestive for CAPS in two different centers (pediatric vs adult). In consideration of the prevalence of this mutation in the general population, 90 healthy individuals were also analyzed for the same mutation.

## Results

the Q703K mutation was found in the 35 screened patients (pediatric 17 vs adult 18, with the mean age was 23,7 years, range 3-64). The mean age at onset was 21,5 years (range 0,5-57). Thirty patients were heterozygous for theQ703K mutation only. Two pts displayed other mutations of NLRP3 gene (M604I in one CINCA and D303N and V198M in a MWS). Three patients display a monoallelic variant of MEFV gene (R202Q, V726A, D303N). The mean follow-up was 2,5 years (range 0,2-8).

The prevalent clinical features were fever and urticarial rash (23 pts), urticarial rash without fever (6 pts) and periodic fever only (6 pts). The main clinical manifestations and treatment are reported in the table. According to the judgment of the physician in charge, a CAPS-like phenotype was observed in 23 patients (66%). In 12 pts (34%) an alternative diagnosis was pointed out (mainly undifferentiated periodic fever). The frequency of the variant Q703K in normal controls was 2,22%.

## Conclusion

The Q703K mutation can be associated to a mild CAPS-like phenotype. Most of the patients do not require anti-IL1 treatment and respond satisfactory to steroid on demand. Even if this variant can be found in patients with a other conditions, the prevalence in patients with CAPS-like phenotype is higher than that detected in healthy controls.

## Disclosure of interest

None declared.

**Table 1:** 

	Fever and Urticarial Skin Rash (23 pts)	Periodic Fever (6 pts)	Urticarial Skin Rash (6 pts)
**Elevation of acute phase reactants**	22	4	2
**Other Symptoms** - Arthralgia -Transient arthritis - Conjunctivitis - Headache - Mental retardation - Dysmorphic bone	22 9 7 9 1 1	2 0 1 2 0 1	3 2 1 1 0 0

**Therapy** - Steroids - NSAIDs - Anti IL-1 Blockers - Others	13 15 6 10	3 3 0 0	5 3 1 3

